# CO_2_ Sequestration in the Production of Portland Cement Mortars with Calcium Carbonate Additions

**DOI:** 10.3390/nano11040875

**Published:** 2021-03-30

**Authors:** Marius-George Parvan, Georgeta Voicu, Alina-Ioana Badanoiu, Adrian-Ionut Nicoara, Eugeniu Vasile

**Affiliations:** Department of Science and Engineering of Oxide Materials and Nanomaterials, Faculty of Applied Chemistry and Material Science, University POLITEHNICA of Bucharest, 1-7 Gh. Polizu Street, District 1, RO-011061 Bucharest, Romania; marius.george.parvan@gmail.com (M.-G.P.); alina.badanoiu@upb.ro (A.-I.B.); adi.nicoara18@gmail.com (A.-I.N.); eugeniu.vasile@upb.ro (E.V.)

**Keywords:** Portland cement, nano-CaCO_3_, limestone filler, carbon footprint reduction, CO_2_ sequestration

## Abstract

The paper presents the obtention and characterization of Portland cement mortars with limestone filler and nano-calcite additions. The nano-calcite was obtained by the injection of CO_2_ in a nano-Ca(OH)_2_ suspension. The resulted nano-CaCO_3_ presents different morphologies, i.e., polyhedral and needle like crystals, depending on the initial Ca(OH)_2_ concentration of the suspension. The formation of calcium carbonate in suspensions was confirmed by X-ray diffraction (XRD), complex thermal analysis (DTA-TG), scanning electron microscopy (SEM) and transmission electron microscopy (TEM and HRTEM). This demonstrates the viability of this method to successfully sequestrate CO_2_ in cement-based materials. The use of this type of nano-CaCO_3_ in mortar formulations based on PC does not adversely modify the initial and final setting time of cements; for all studied pastes, the setting time decreases with increase of calcium carbonate content (irrespective of the particle size). Specific hydrated phases formed by Portland cement hydration were observed in all mortars, with limestone filler additions or nano-CaCO_3_, irrespective of curing time. The hardened mortars with calcium carbonate additions (in adequate amounts) can reach the same mechanical strengths as reference (Portland cement mortar). The addition of nano-CaCO_3_ in the raw mix increases the mechanical strengths, especially at shorter hardening periods (3 days).

## 1. Introduction

In recent years there has been a constant interest from political and scientific community in identifying new ways to reduce greenhouse gases to combat global warming and climate change. The cement industry is one of the major contributors to the world’s CO_2_ emissions; it is generally accepted that the production of one ton of cement (CEM I) can release up to one ton of carbon dioxide [1,2]. 

Portland cement is obtained by the grounding of clinker with gypsum and other admixtures. The clinker is produced by heating a raw mix, consisting mainly of limestone and clay (marl) up to 1450 °C in a rotary kiln. Several chemical processes take place in the rotary kiln and transform the raw mix in the clinker; limestone calcination is the main chemical process which generates up 60–65% CO_2_ of cement manufacturing. CO_2_ emissions are also generated by the burning of fuels used to heat the rotary kiln [3]. 

Portland cement is one of the key ingredients of concrete, which is the third most used substance in the world after air and water [4]. As a result, the reduction in the carbon footprint of cement production has drawn a great deal of attention from scientists around the globe in a unified effort to preserve our planet. One of the currently studied methods for the achievement of a greener cement production is carbon dioxide sequestration in cement-based materials [1,2,3,4,5]. 

The carbonatation of concrete mixtures produced with various types of cements represent a new approach aiming to sequestrate CO_2_ in concrete. Microstructural and mechanical properties of cement-based materials may be altered by early-age carbonation which often provides hydrates with significant differences [6,7,8,9,10,11,12,13,14,15,16,17,18,19].

A great deal of research has been performed in order to substantiate the use of finely ground limestone (limestone filler -L) as a partial substitute for Portland cement in the attempt to reduce the amount of clinker used in the production of Portland cement and consequently to reduce the green gas emissions associated with this process [20,21]. According to Panesar and Zhang [21], for lower replacement levels (up to 10%) concrete or mortar with limestone filler has similar properties compared with control mix, but the increase of substitution ratio can affect properties such as strength, porosity and permeability. To mitigate these drawbacks research studies were performed using nano-CaCO_3_ [22,23,24,25,26,27,28,29,30,31,32,33,34,35]. Ge et al. [32] reported that the addition to mortar/concrete of nano-calcium carbonate (in adequate amounts) increases the compressive strength and reduces average pore diameter. 

Qin et al. [2] proposed a new technology aiming to reduce the carbon footprint of the cement industry, consisting mainly in the use of the CO_2_ generated in cement production to carbonate suspensions of lime with concentrations of 0.17%, 0.34% and 1.02% by mass. The introduction of the resulting nano-calcium carbonate in cement paste improved the mechanical properties and refined the pore structure. 

This study assesses the feasibility of producing calcium carbonate suspensions with higher concentrations (0.3% up to 5% nano-CaCO_3_) by the carbonation of nano-CaO suspensions and to compare their influence on the main properties of Portland cement mortars with the effect determined by limestone filler additions.

## 2. Materials and Methods

### 2.1. Materials

The following materials were used in this study:

- calcium oxide nano-powder (<160 nm BET particle size, Sigma Aldrich, Darmstadt, Germany) and CO_2_ gas, with 100% concentration;
- Portland cement (PC) CEM I 42.5R, with 3095 cm^2^/g Blaine specific surface area and oxide composition presented in Table 1; the PCs oxide composition was assessed by the chemical methods described in European standard EN 196-2:2013 [36];
- limestone filler (L) with 97 wt.% CaCO_3_ and a fineness corresponding to 5350 cm^2^/g Blaine specific surface area.

### 2.2. Preparation of Cement Pastes and Mortars with Limestone Filler and Nano-CaCO_3_

Ca(OH)_2_ aqueous suspension (aq. susp.) in various concentrations (0.5%, 3.7% and 7.4%) were prepared by partially dissolution of nano-CaO in water. The next step consists of CO_2_ gas injection in Ca(OH)_2_ aq. susp., at a rate of 15 dm^3^/min. The duration of injection operation was 3 minutes for each liter of Ca(OH)_2_ aq. susp. In order to produce a homogenous suspension by the reaction of CO_2_ with nano-Ca(OH)_2_, the suspensions were continuously stirred with a magnetic stirrer. The pH of Ca(OH)_2_ aqueous suspensions (before CO_2_ injection) was 12.5 and after the CO_2_ injection the pH dropped to 6.5, which suggests the complete carbonation of nano-Ca(OH)_2_ in the suspension. The density (assessed with a hydrometer) was 1 g/cm^3^ for all suspensions. The aqueous suspensions where vigorously stirred and used to produce cement pastes and mortars. 

Table 2 shows the composition of the studied cements prepared with limestone filler or carbonated nano-Ca(OH)_2_ aqueous suspensions. 

The amount of nano-CaCO_3_ was calculated based on the concentration of the aqueous suspensions and the amount of liquid (aq. susp.) used to achieve a liquid to cement ratio of 0.5.

The codification of samples (pastes or mortars) with Ca(OH)_2_ carbonated aq. susp. was made according to the equivalent dosage of calcium hydroxide, i.e., CC0.5—with 0.5% Ca(OH)_2_ carbonated aq. susp.; CC3.7—with 3.7% Ca(OH)_2_ carbonated aq. susp.; CC7.4—with 7.4% Ca(OH)_2_ carbonated aq. susp. 

Mortars were prepared using the cement compositions presented in Table 2, aggregate and water. The cement to aggregate ratio was 0.3 and water to cement ratio = 0.5. The aggregate was CEN standard siliceous sand with the grading fully complying with EN 196-1:2016, Part 1 [37]. The fresh mortars were cast in prism molds (160 × 40 × 40 mm) and compacted by impact. The resulting three mortar specimens (for each composition) were cured in humid environment (R.H. 95%) for 3, 7 and 28 days.

### 2.3. Methods

The mineralogical composition of materials was assessed by X-ray diffraction (XRD), with a Shimadzu XRD 6000 diffractometer (Shimadzu, Kyoto, Japan), with Ni-filtered CuKα radiation (α = 1.5406 Å), 2θ = 5–65°. 

The morphology and microstructure of the materials (nano-CaCO_3_ and hardened PC mortars with/without limestone or nano-CaCO_3_ content) were assessed by electron microscopy-scanning electron microscopy (SEM) and transmission electron microscopy (HR-TEM). SEM analysis were performed using a Quanta Inspect F scanning electron microscope (FEI Company, Hillsboro, OR, USA) with a Schottky emission electron beam (1.2 nm resolution at 30 kV and 3 nm at 1 kV for BSE; gold was used for the coating of specimens). The TEM analyses were performed using a TecnaiTM G2 F30 S-TWIN high resolution transmission electron microscope (HR-TEM) (Thermo Fisher—former FEI, Eindhoven, Nederland) equipped with STEM-HAADF detector, EDX and EELS. The average particle size and crystallinity degree were estimated using this method.

The water for standard consistency and setting time of cement pastes was assessed with the methods presented in European standard EN 196—1:2016, Part 3 [38]. 

Flexural and compressive strength were assessed using a Matest machine ((MATEST, Treviolo, Italy), in accordance with the method presented in European standard EN 196—1:2016, Part 1 [37]; the strength values represent the average of at least three strength values assessed on mortar specimens cured in similar conditions.

## 3. Results and Discussion

**Figure 1:**
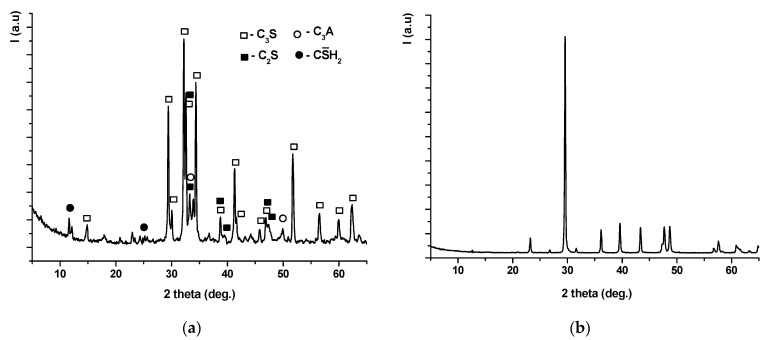
X-ray diffraction pattern of Portland cement (**a**), and of limestone filler (**b**).

The mineralogical compositions of Portland cement and limestone filler were assessed by XRD. On the XRD pattern of Portland cement (Figure 1a) the following peaks are present: alite (Ca_3_SiO_5_ or C_3_S, JCPDS 31-0301), belite (Ca_2_SiO_4_ or C_2_S, JCPDS 31-0299), tricalcium aluminate (Ca_3_Al_2_O_6_ or C_3_A, JCPDS 38-1429), and gypsum (CaSO_4_·2H_2_O or C$\bar{S}$H_2_, JCPDS 21-0816). For the limestone filler (Figure 1b) the main phase assessed by XRD is calcite (CaCO_3_, JCPDS 72-1650).

**Figure 2:**
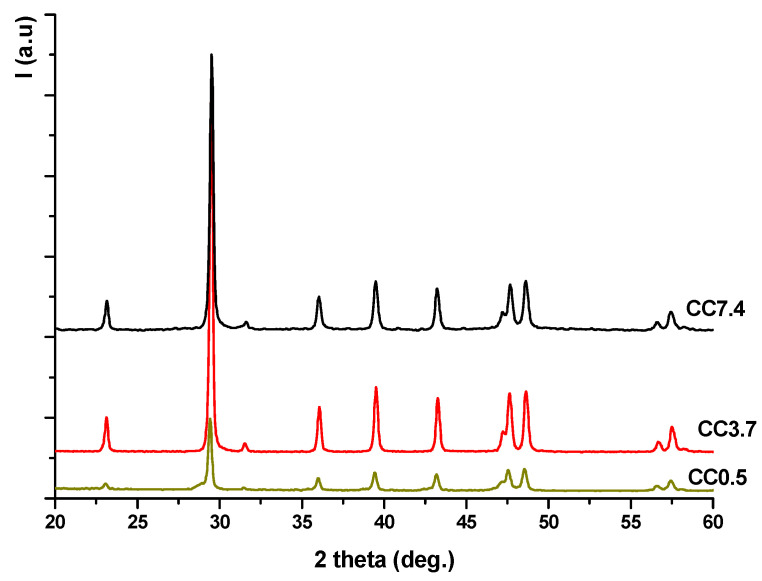
X-ray diffraction patterns of dried powders.

To confirm the presence of calcium carbonate in aq. susps., 1 ml from each suspension (vigorously stirred in order to be homogenous) was sampled and dried at 50 °C up to constant weight. The XRD patterns of the resulting powders (Figure 2) showed the presence of a single crystalline compound—CaCO_3_ (JCPDS 83-1762).

**Figure 3:**
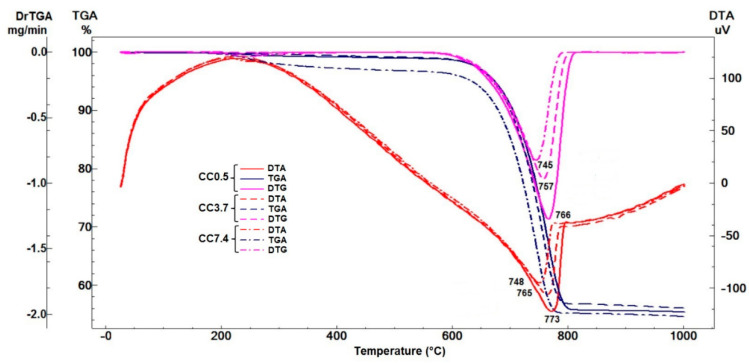
Thermal analysis of dried powders CC0.5, CC3.7 and CC7.4.

The differential thermal analysis (DTA) curves of dried powders (Figure 3) show an intense endothermic effect with maximum at 748–773 °C, with a corresponding weight loss assessed on TG curves between 650–800 °C; this effect is attributed to the CaCO_3_ decarbonation [39] and is in good correlation with the results obtained by XRD analysis, previously presented. Because decarbonation process occurs below 800 °C, it is suggested that carbonate phases are microcrystalline [39].

**Figure 4:**
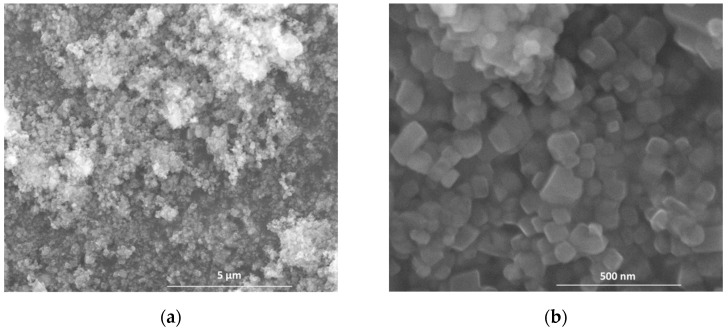

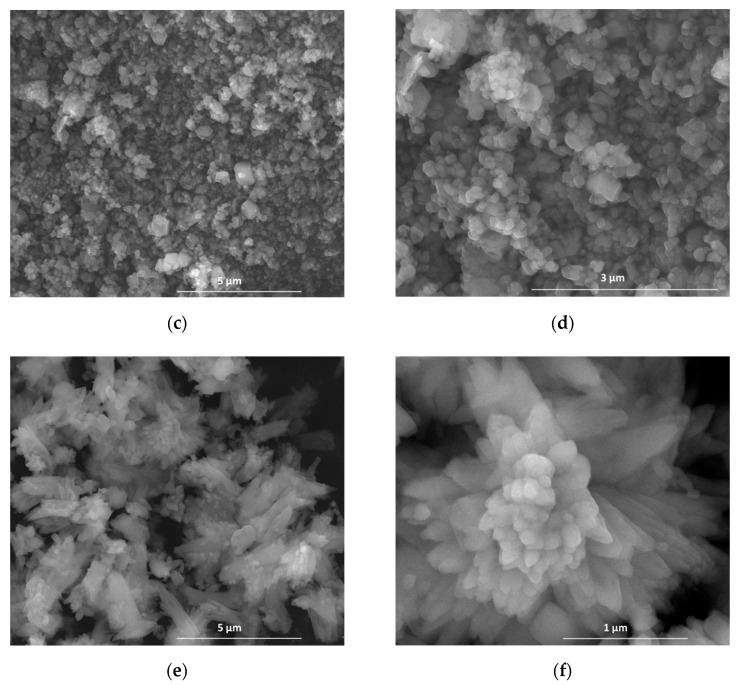
Scanning electron microscopy (SEM) images of dried powders CC0.5 (**a**,**b**), CC3.7 (**c**,**d**) and CC7.4 (**e**,**f**).

As presented in Figure 4, calcium carbonate powders have different morphologies which can be correlated with their concentration in aq. susp. One can observe the presence of CaCO_3_ polyhedral crystals in the SEM images (Figure 4a–d) of the powders as a result of the drying of carbonated aq. susp. with 0.5% and 3.7% Ca(OH)_2_; these crystals are the result of the Ca(OH)_2_ carbonation. The size of CaCO_3_ crystals increases with the increase of the initial concentration of Ca(OH)_2_ aq. susp; for an initial concentration of 7.4% Ca(OH)_2_ in aq. susp. the resulted CaCO_3_ crystals have elongated shapes and forms flower-like aggregates (Figure 4e,f). These shapes (polyhedral or elongated) of CaCO_3_ crystals were also assessed in other studies [40,41,42]. These results are in good correlation with those obtained by XRD and DTA-TG analyses.

**Figure 5:**
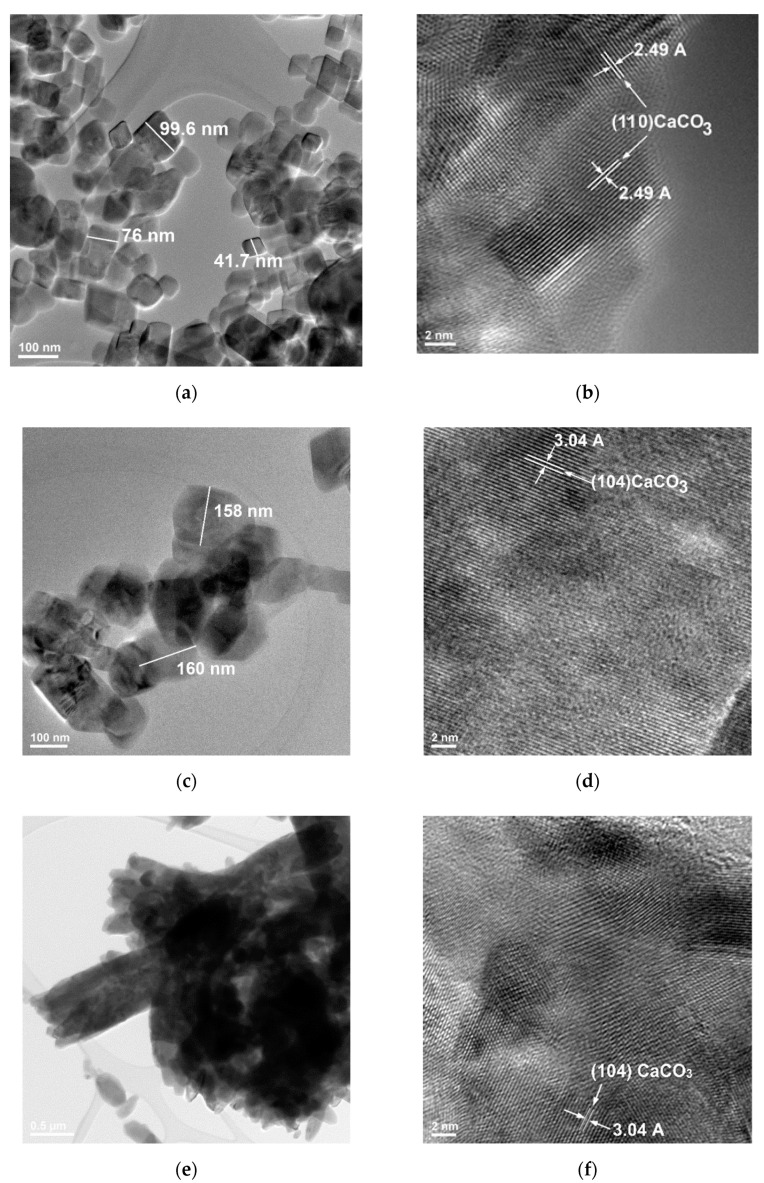
Transmission electron microscopy (TEM) (**a**,**c**,**e**) and HRTM (**b**,**d**,**f**) images of dried powders CC0.5 (**a**,**b**), CC3.7 (**c**,**d**) and CC7.4 (**e**,**f**).

The morphology of calcium carbonate crystals was also assessed by high resolution transmission electron microscopy (HR-TEM), as shown in Figure 5. This method permits an assessment of the total conversion of Ca(OH)_2_ nano-grains in calcium carbonate, irrespective of the initial concentration in nano-CaO of the starting suspension (Figure 5b,d,f); the size of crystals assessed by HR-TEM starts in tens of nanometers (for CC0.5—Figure 5a) and increases with the initial concentration of Ca(OH)_2_ aq. susp., reaching hundreds of nanometers for CC3.4 and CC7.4 (Figure 5c,e).

**Figure 6:**
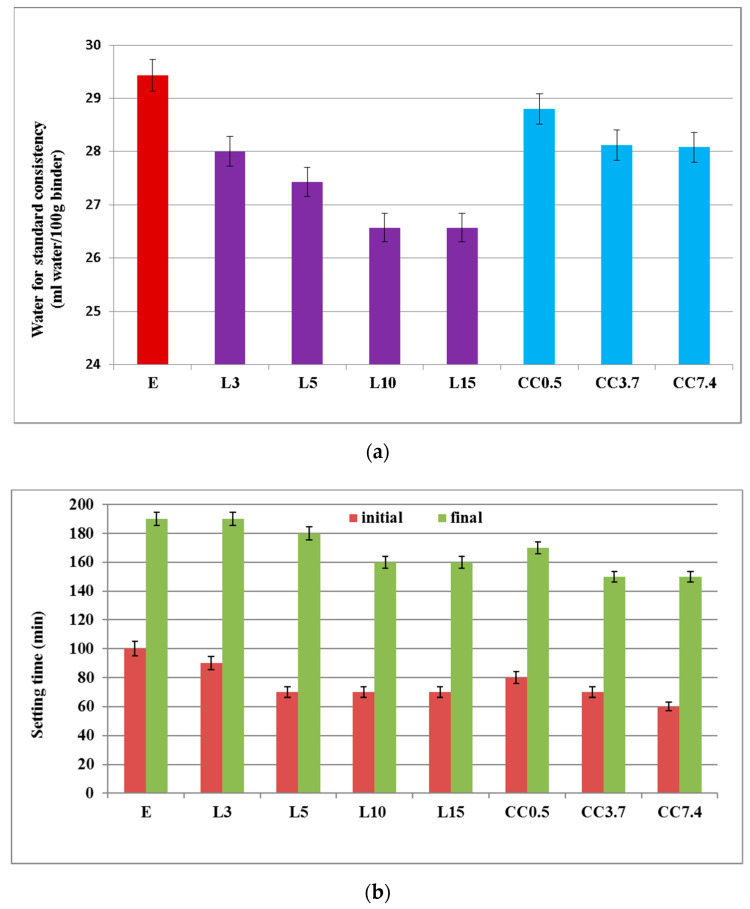
Water for standard consistency (**a**) and setting time (**b**) of binding pastes.

The values of water for standard consistency of pastes decrease (with respect to reference—PC paste) with the increase of limestone filler (L) content (Figure 6a). Yahia et al. [43] noticed a similar behaviour for Portland cements with limestone filler additions, i.e., for a given water to cement ratio, there is an optimum value of powder content that can ensure suitable fresh properties of the mixture. These authors explain this by a physical effect determined by limestone filler particles which are smaller compared with those of Portland cement and fill the voids existent between them; this reduces the interparticle friction and liberates part of the mixing water otherwise entrapped in the system, thus increasing the fluidity of the fresh paste. For the specimens with nano-CaCO_3_, one can also notice the decrease of water for standard consistency with the increase in the nano-CaCO_3_ amount (with reference to PC paste), but the values of water for standard consistency are higher compared with those assessed for cement with limestone content (see, for example, the cements with 5% limestone filler L5 and 5% nano CaCO_3_ CC7.4). This is in good agreement with the previously mentioned explanation, given the much smaller dimensions of this type of calcium carbonate compared with the limestone particles.

For all studied pastes, the setting time decreases with the increase of calcium carbonate content (Figure 6b); the CaCO_3_ particles act as crystallization sites for the newly formed hydrates (in the reaction of PC with water). The smaller values for final setting time assessed for the composition with nano-carbonate (see for example CC7.4 compared with L5) can be also due to the high fineness of calcium carbonate (nano powder).

**Figure 7:**
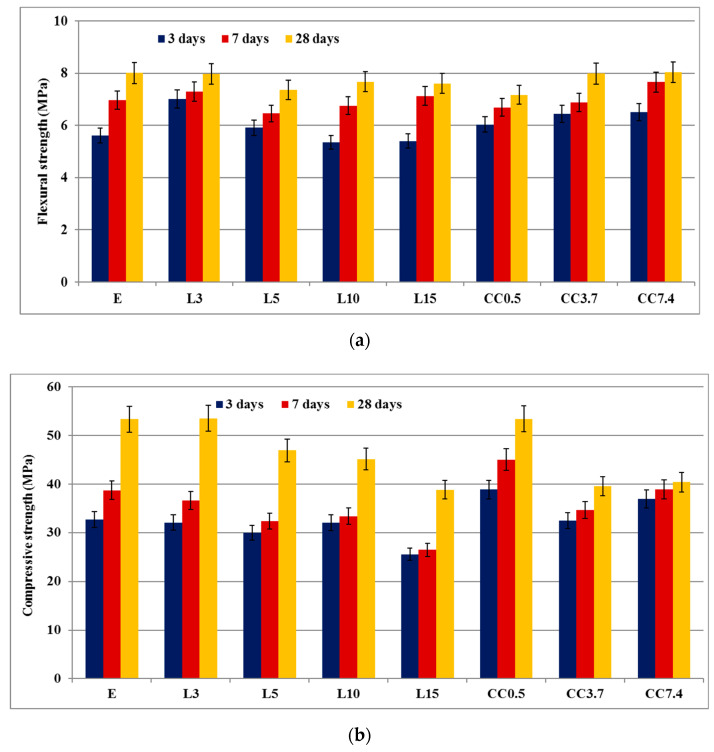
Mechanical strengths of mortars versus hardening time: flexural strength (**a**) and compressive strength (**b**).

The evolution of mechanical strengths was assessed on mortars hardened for 3, 7 and 28 days—Figure 7. As expected, both compressive and flexural strengths increase with the increase in curing time from 3 to 28 days, due to the development of PC hydration and the hardening process. The substitution of PC with 3% and 5% limestone filler does not substantially modify the mechanical strengths with reference to E (PC), but the increase in substitution rate has a negative influence especially after 28 days of hardening. This is explained by the dilution effect of cement—limestone filler substitutes for the PC which is the active component of the binder (see Table 2).

The addition of nano-CaCO_3_ in the raw mix, increases the mechanical strength of the mortars, especially at shorter periods of time (3 and 7 days). The important increase of compressive strengths assessed for CC0.5 (with reference to E) can be explained by an adequate microstructure (refined pore structure) in which a higher amount of hydrates are formed (due to the nucleation effect determined by the CaCO_3_ nanoparticles) which results in the reduction of the porosity.

**Figure 8:**
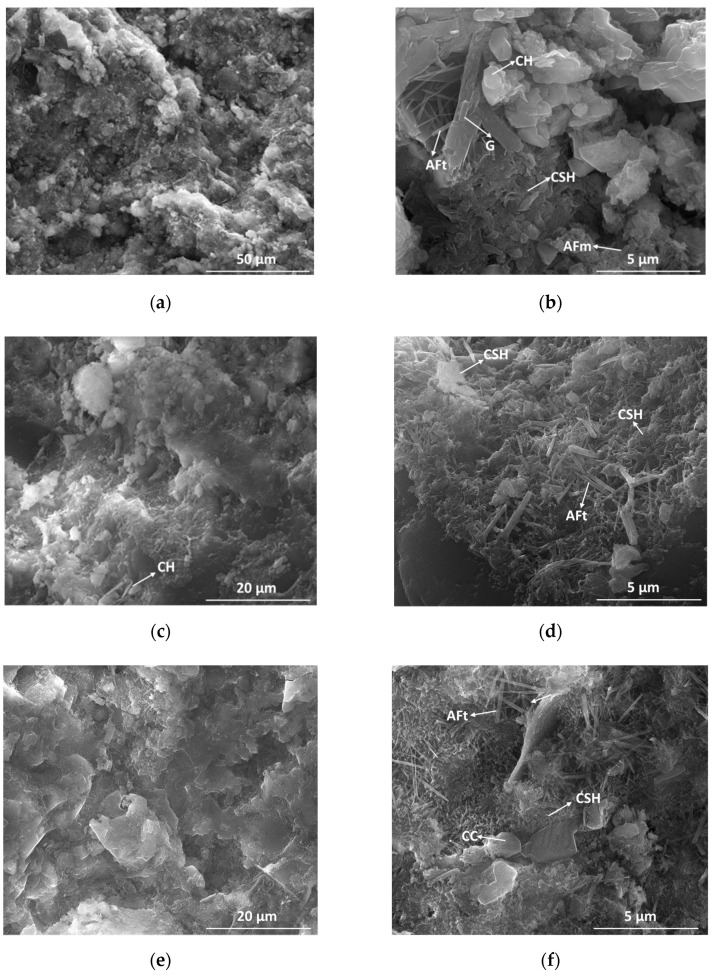

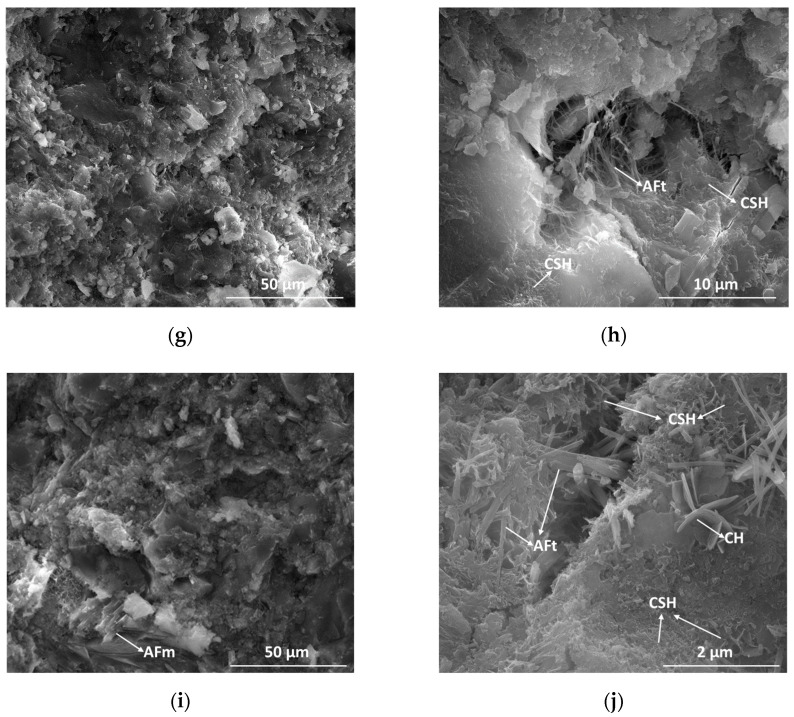
SEM images of hardened mortars at 28 days: E (**a**,**b**), L3 (**c**,**d**), L15 (**e**,**f**), CC0.5 (**g**,**h**) and CC7.4 (**i**,**j**).

Figure 8 shows the SEM images of mortars cured for 28 days. The main phases assessed by this method are: hexagonal plate-like crystals attributed to Ca(OH)_2_ (CH), fine needles and films attributed to calcium silicate hydrates (CSH), well defined needle-shaped formations characteristic of ettringite (AFt), monoclinic gypsum (G) crystals, parallel plates forming a beam characteristic of the calcium mono-sulfate aluminate hydrate (AFm) phase and polyhedral particles of calcium carbonate (CC). 

This method does not permit the assessment of any new hydration products when the calcium carbonate additions (with different grain sizes) are used as additions to Portland cement.

## 4. Conclusions

The experimental results presented in this study demonstrate that carbon dioxide can be successfully sequestered in cement-based materials. 

CO_2_ sequestration in nano-CaCO_3_ was obtained by the injection of CO_2_ in a nano-Ca(OH)_2_ suspension. The resulting nano-CaCO_3_ particles present different morphologies, i.e., polyhedral and needle like crystals, depending on the initial Ca(OH)_2_ concentration of the suspension. 

The use of this type of nano-CaCO_3_ in Portland cement pastes determines a decrease in the water for standard consistency with reference to PC paste, but the values of water for standard consistency are higher as compared with those assessed for the cement with limestone filler (in the same amount); this is due to the much smaller dimensions of this nano-calcium carbonate compared with the limestone particles.

The use of this nano-carbonate does not adversely modify the initial and final setting time of cements; for all studied pastes, the setting time decreases with the increase of calcium carbonate content (irrespective of the particle size). 

Specific hydrated phases formed by Portland cement hydration were observed in all mortars, with limestone filler additions or nano-CaCO_3_, irrespective of curing time. 

The hardened mortars with calcium carbonate additions (in adequate amounts) can reach the same mechanical strengths as reference (Portland cement mortar). The addition of nano-CaCO_3_ in the raw mix increases the mechanical strengths, especially at shorter hardening periods (3 days).

## Figures and Tables

**Table 1:** Oxide composition of Portland cement.

**Component**	CaO	SiO_2_	Al_2_O_3_	Fe_2_O_3_	MgO	SO_3_	LOI
**Content (wt.%)**	63.78	20.12	4.58	3.99	1.20	2.61	3.72

LOI—loss on ignition.

**Table 2:** Composition of the studied cements.

Cement	E	L3	L5	L10	L15	CC0.5	CC3.7	CC7.4
**Portland cement (wt.%)**	100	97	95	90	85	99.65	97.5	95
**Limestone filler (wt.%)**	0	3	5	10	15	0	0	0
**Nano-CaCO_3_ (wt.%)**	0	0	0	0	0	0.35	2.5	5

## Data Availability

Not applicable.
